# Outcome of Prolonged Ventricular Fibrillation and CPR in a Rat Model of Chronic Ischemic Left Ventricular Dysfunction

**DOI:** 10.1155/2013/564501

**Published:** 2013-12-17

**Authors:** Xiangshao Fang, Lei Huang, Shijie Sun, Max Harry Weil, Wanchun Tang

**Affiliations:** ^1^Department of Emergency Medicine, Sun Yat-sen Memorial Hospital, Sun Yat-sen University, Guangzhou, Guangdong 510120, China; ^2^Weil Institute of Critical Care Medicine, Rancho Mirage, CA 92270, USA; ^3^The Keck School of Medicine of the University of Southern California, Los Angeles, CA 90033, USA

## Abstract

Patients with chronic left ventricular (LV) dysfunction are assumed to have a lower chance of successful CPR and lower likelihood of ultimate survival. However, these assumptions have rarely been documented. Therefore, we investigated the outcome of prolonged ventricular fibrillation (VF) and CPR in a rat model of chronic LV dysfunction. Sprague-Dawley rats were randomized to (1) chronic LV dysfunction: animals underwent left coronary artery ligation; and (2) sham control. Echocardiography was used to measure cardiac performance before surgery and 4 weeks after surgery. Four weeks after surgical intervention, 8 min of VF was induced and defibrillation was delivered after 8 min of CPR. LV dilation and low ejection fraction were observed 4 weeks after coronary ligation. With optimal chest compressions, coronary perfusion pressure values during CPR were well maintained and indistinguishable between groups. There were no differences in resuscitability and numbers of shock required for successful resuscitation between groups. Despite the significantly decreased cardiac index in LV dysfunction animals before induction of VF, no differences in cardiac index were observed between groups following resuscitation, which was associated with the insignificant difference in postresuscitation survival. In conclusion, the outcomes of CPR were not compromised by the preexisting chronic LV dysfunction.

## 1. Introduction

A majority of episodes of sudden cardiac deaths occur in victims with ischemic heart disease. Ischemic heart disease may develop over a lengthy span of time and is often associated with left ventricular (LV) remodeling. This ultimately leads to chronic ischemic LV dysfunction with subsequent congestive heart failure. Lower ejection fraction (EF) has been consistently demonstrated to be the strongest independent predictor of sudden cardiac death [1–3]. When cardiac arrest occurs in patients with chronic ischemic LV dysfunction, they are assumed to have a lower chance of successful cardiopulmonary resuscitation (CPR) and lower likelihood of ultimate survival. However, these assumptions have rarely been documented. Little is known about prognostic information concerning the outcomes of CPR in patients with chronic ischemic LV dysfunction.

The goals of the present study were therefore to obtain prognostic information on the outcome of prolonged ventricular fibrillation (VF) and CPR in the chronic ischemic LV dysfunction due to complete left coronary artery ligation in Sprague-Dawley rats. We hypothesized that when undergoing prolonged VF/CPR, chronic ischemic LV dysfunction animals would be less likely to be resuscitated. If resuscitated, such animals would be likely to have more severe postresuscitation myocardial dysfunction and decreased duration of postresuscitation survival.

## 2. Materials and Methods

This study was approved by the Institutional Animal Care and Use Committee of the Weil Institute of Critical Care Medicine. All animals received humane care in compliance with the *Principles of Laboratory Animal Care *formulated by the National Society for Medical Research and the *Guide for the Care and Use of Laboratory Animals* prepared by the Institute of Laboratory Animal Resources and published by the National Institutes of Health.

### 2.1. Study Design

Fourteen male Sprague-Dawley rats weighing 500 ± 50 g were randomized into (1) chronic ischemic LV dysfunction group (*n* = 7): the animals underwent left coronary artery ligation 4 weeks before induction of VF; and (2) control group (*n* = 7). The animals received sham operation without coronary artery ligation 4 weeks before induction of VF.

### 2.2. Chronic Ischemic LV Dysfunction Model

The animals were fasted overnight except for free access to water. They were anesthetized by intraperitoneal injection of pentobarbital (45 mg/kg). The animals were then orally intubated and mechanically ventilated with room air. Electrocardiogram (ECG) was continuously monitored. After measurements of baseline myocardial function using noninvasive transthoracic echocardiography, a thoracotomy via the third left intercostal space was performed. The atrial appendage was elevated and the left coronary artery near its origin was ligated. Successful ligation was confirmed by the ST segment elevation. The chests were then closed, and the animals were returned to their cages. Postsurgical pain was controlled with intramuscular injection of ketorolac (0.4 mg/kg). Control rats were prepared similarly except that the coronary artery was not ligated.

### 2.3. Experimental Procedures of VF/CPR

Four weeks after surgical intervention, the animals were reanesthetized and intubated. Cardiac geometry and function were assessed by echocardiography. A PE-50 catheter (Becton Dickinson) was advanced from the right carotid artery into the left ventricle for measurement of LV pressure. A PE-50 catheter was advanced through the left external jugular vein into the right atrium for measurement of right atrial pressure. For electrical induction of VF, a 4 French PE catheter was advanced through the right external jugular vein into the right atrium, and through its lumen a precurved guide wire was then advanced into the right ventricle for electrically inducing VF. A PE-50 catheter was advanced through the left femoral artery into the thoracic aorta for measurement of mean aortic pressure (MAP). A thermocouple microprobe (9030-12-D-34, Columbus Instruments; Columbus, OH) was advanced from the right femoral artery into the descending thoracic aorta for measurement of blood temperature. ECG was recorded. A heat lamp was used to maintain body temperature at 36.8°C (±0.2%).

The animals were mechanically ventilated with room air at a tidal volume of 0.55 mL/100 g and a frequency of 100 breaths/min. A progressive increase in 60 Hz current to a maximum of 4 mA was then delivered to the right ventricular endocardium. The current flow was continued for 3 min to preclude spontaneous reversal of VF. Mechanical ventilation was discontinued after onset of VF. Precordial compression was then begun and mechanical ventilation with 100% O_2_ was resumed 8 min after the onset of VF. Precordial compression at a rate of 200 min^−1^ was synchronized to provide a compression/ventilation ratio of 2 : 1. The depth of compression was adjusted to maintain a coronary perfusion pressure (CPP) at 24 ± 2 mmHg. Resuscitation was attempted with up to three 2-J biphasic waveform countershocks (CodeMaster XL, Heartstream Operation, Philips; Seattle, WA) after 8 min of CPR. Return of spontaneous circulation (ROSC) was defined as an organized rhythm with MAP ≥60 mmHg for ≥5 min.

Following ROSC, the animals were monitored for 4 hours. All catheters were then removed. The animals were observed for an additional 68 hours after which they were euthanized with an intraperitoneal injection of pentobarbital sodium (150 mg/kg). An autopsy was performed to confirm the complete ligation of left coronary artery, and organs were inspected for gross abnormalities, including evidence of traumatic injuries consequent to cannulation, airway management, or precordial compression.

### 2.4. Measurements

LV geometry and cardiac function prior to and 4 weeks after ligation was quantitated with a Sonos 2500 echocardiographic system utilizing a 7.5 Hz transducer (Model 21363A, Hewlett-Packard Co., Medical Products Group, Andover, MA). The animal hearts were imaged in the parasternal short-axis plane through the anterior chest. At two-dimensional imaging of short-axis view, left ventricular end-systolic volumes (LVESV) and left ventricular end-diastolic volumes (LVEDV) were calculated by the method of discs (Acoustic Quantification Technology, Hewlett-Packard, Andover, MA). From these, EF was computed.

Aortic, LV, right atrial pressures, and ECG were recorded via a WinDaqdata-acquisition system (DataQ; Akron, OH). CPP was calculated as the difference between aortic and time-coincident right atrial pressures in the interval between chest compressions.

Myocardial function during VF/CPR experimental phase was assessed from measurements of LV pressure and cardiac output. The rate of LV pressure increase at 40 mmHg (*dP*/*dt*_40_) was measured by analog differentiation as an indicator of isovolumic contractility. The rate of LV pressure decline (−*dP*/*dt*) was measured as an indicator of myocardial relaxation. Cardiac output was measured by a thermodilution technique with the aid of a cardiac-output computer fabricated at our institute. The data were reported as cardiac index (CI) values as an indicator of global pump function.

### 2.5. Analyses

Measurements are reported as means ± SD. Comparisons between groups before surgical operation and 4 weeks after coronary ligation were performed by using Student's *t*-test. Following ROSC, comparisons between time-based measurements within each group were performed with analysis of variance for repeated measurements. The success of resuscitation and 72-hour survival rate were analyzed with Fisher's exact test. Survival analysis was performed with the Kaplan-Meier method. A value of *P* < 0.05 was regarded as significant.

## 3. Results

**Figure 1:**
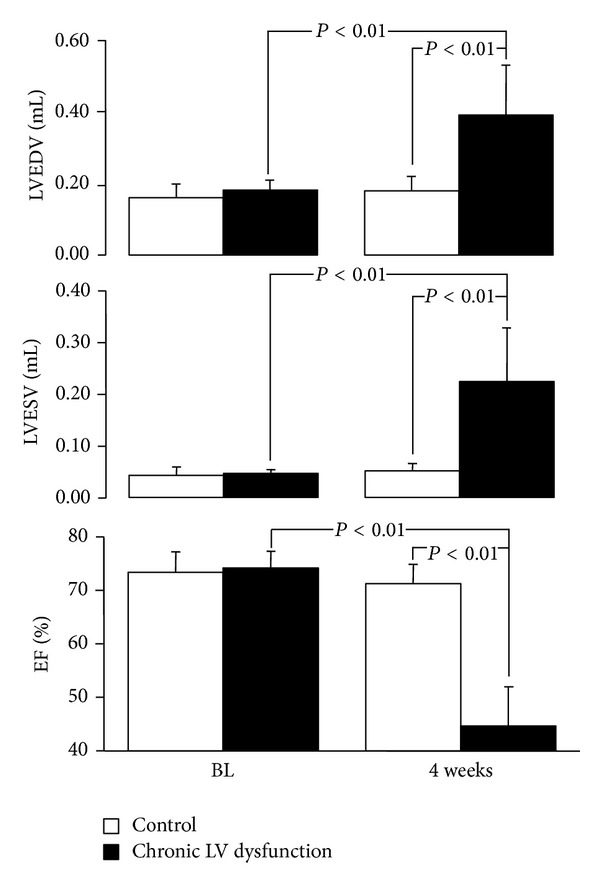
Echocardiographic measurements at baseline (BL) and 4 weeks after left coronary artery ligation. Values are means ± SD. LVEDV: left ventricular end-diastolic volume; LVESV: left ventricular end-systolic volume; EF: ejection fraction.

Before surgical operations, there were no differences in baseline values of echocardiographically measured LVEDV, LVESV, and EF between groups (Figure 1). Significant decreases in EF and increases in LVEDV and LVESV were documented in chronic ischemic LV dysfunction animals 4 weeks after coronary ligation.

CPP values were maintained at 24 ± 2 mmHg for all animals during the entire period of CPR. There were no differences in resuscitability and total shock energy required for ROSC between groups (Table 1).

**Figure 2:**
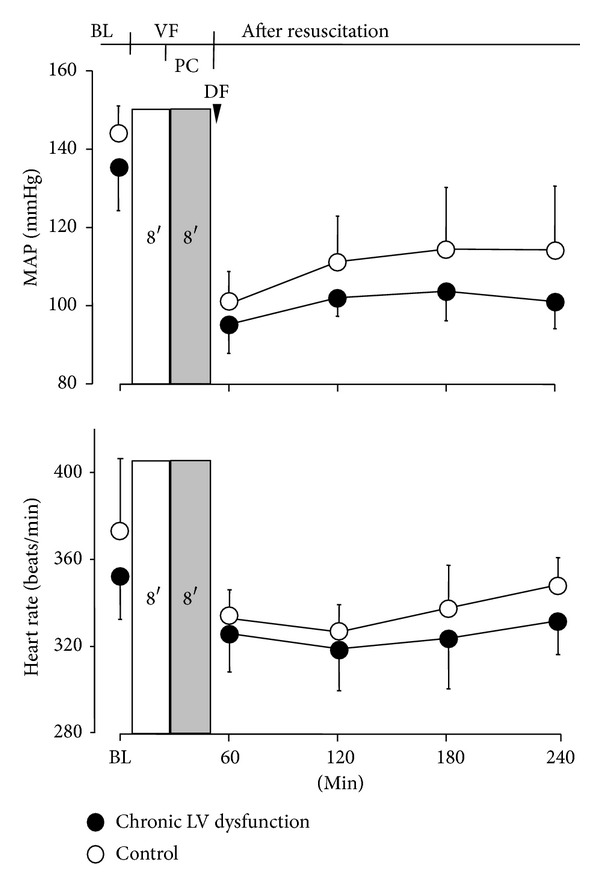
Mean aortic pressure and heart rate before onset of cardiac arrest and following resuscitation. Values are means ± SD. MAP: mean aortic pressure; BL: baseline; VF: ventricular fibrillation; PC: precordial compression; DF: defibrillation.

Before induction of VF and following ROSC, there were no significant differences in both MAP and heart rate in heart failed animals when compared with control animals (Figure 2).

**Figure 3:**
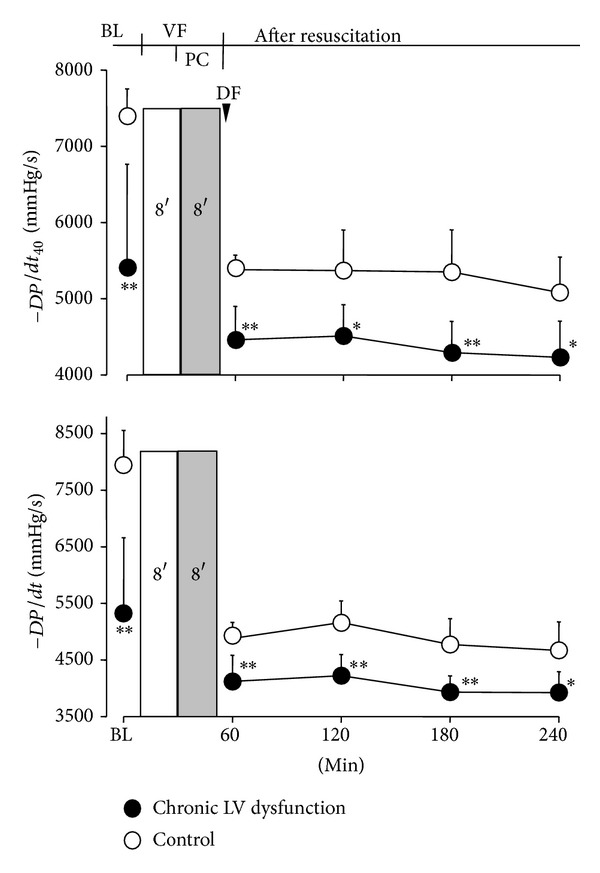
*dP*/*dt*_40_ and −*dP*/*dt* before onset of cardiac arrest and following resuscitation. Values are means ± SD; *P < 0.05; **P < 0.01 versus control. BL: baseline; VF: ventricular fibrillation; PC: precordial compression; DF: defibrillation.

**Figure 4:**
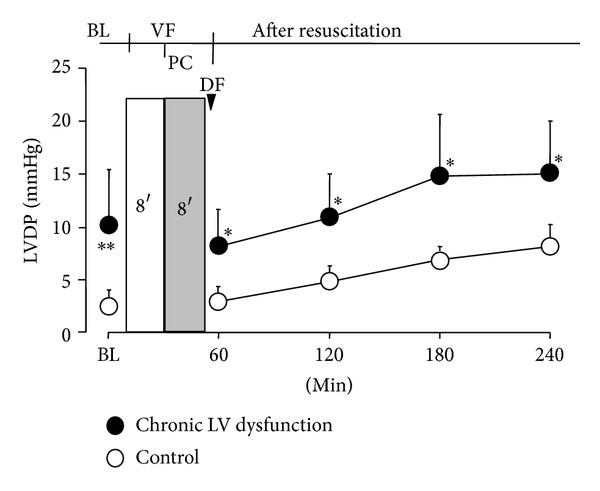
LV end-diastolic pressure (LVDP) before onset of cardiac arrest and following resuscitation. Values are means ± SD; *P < 0.05; **P < 0.01 versus control. BL: baseline; VF: ventricular fibrillation; PC: precordial compression; DF: defibrillation.

**Figure 5:**
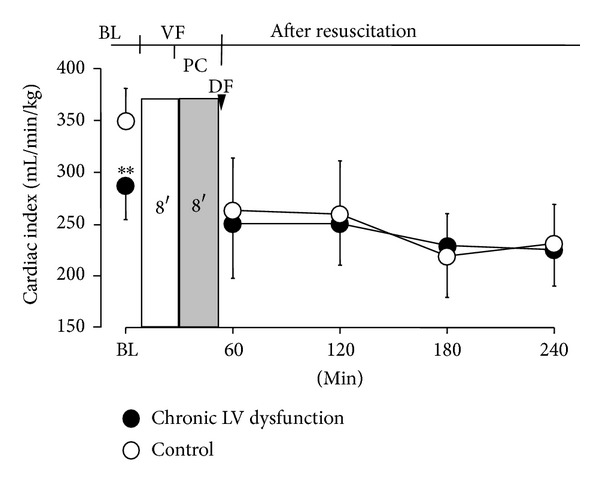
Cardiac index before onset of cardiac arrest and following resuscitation. Values are means ± SD; **P < 0.01 versus control. BL: baseline; VF: ventricular fibrillation; PC: precordial compression; DF: defibrillation.

**Figure 6:**
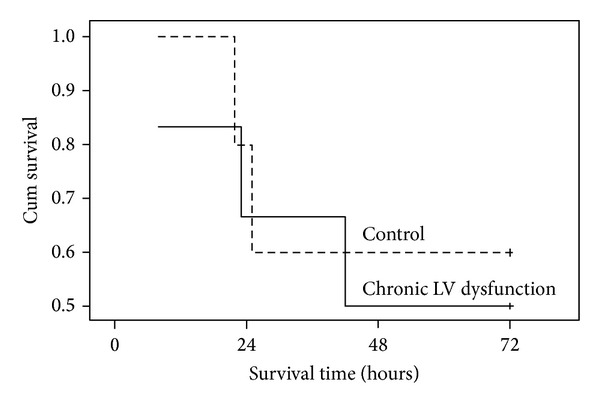
Kaplan-Meier survival curves.

Myocardial function, as measured by *dP*/*dt*_40_ and −*dP*/*dt*, was significantly decreased in LV dysfunction animals before induction of VF and over 4 hours after resuscitation compared with control animals (Figure 3). Similarly, LV end-diastolic pressure (LVDP) was significantly increased in LV dysfunction animals at baseline before induction of VF and following ROSC compared with control animals (Figure 4). Four weeks after coronary ligation but prior to induction of VF, the resting CI in LV dysfunction animals was significantly lower than that of control animals. However, no significant difference in CI was observed between groups following resuscitation (Figure 5). No differences in the number of animals surviving 72 hours and duration of survival (survival curve) were observed between groups (Table 1; Figure 6).

At autopsy, transmural scar formation of the LV anterior wall was observed in LV dysfunction animals. No gross abnormalities were observed at autopsy in any animals.

## 4. Discussion

In this study, CPR was performed effectively after prolonged VF in a rat model of chronic ischemic LV dysfunction. With optimal chest compressions, the ease of defibrillation and cardiac resuscitability were not compromised by the preexisting chronic LV dysfunction. Furthermore, this study revealed that it was the systemic blood that flows through the circulation following ROSC, rather than the preexisting chronic LV dysfunction, which was the predominant determinant of postresuscitation survival.

Fewer studies have directly evaluated the influence of preexisting chronic ischemic LV dysfunction on the likelihood of resuscitability and ultimate postresuscitation survival. Previously we have demonstrated the feasibility of applying CPR in a rat model of chronic nonocclusive left coronary artery constriction [4]. It is unexpected to notice that no differences in resuscitability and postresuscitation short-term outcome were observed between coronary constriction animals and control animals. We suppose that this may be due to the possibilities that heart function is less impaired after coronary artery narrowing and that downtime of VF/CPR is too short to differentiate the effects of myocardial ischemia on the survival outcome [4]. The goals of the present study were therefore to obtain prognostic information on the outcome of prolonged VF/CPR in chronic ischemic LV dysfunction due to left coronary artery complete ligation.

Overt LV dysfunction and extensive ventricular remodeling were observed in left coronary artery ligation animals. Myocardial function as assessed by EF, CI, *dP*/*dt*_40_, and −*dP*/*dt* was significantly depressed and LVDP was significantly increased 4 weeks after coronary ligation. The LV remodeling was manifested by the significantly larger LVEDV and LVESV. Taken together, our results suggested the severe deterioration in LV pump dynamics and extensive ventricular remodeling in this rat model.

In the current study, the number of defibrillations and resuscitability in LV dysfunction animals did not differ from thoat in control. It is well known that the myocardial blood flow is the overriding determinant for the success of resuscitation effort, especially when the duration of untreated cardiac arrest is prolonged [5, 6]. It has also been suggested that CPP correlates well with myocardial blood flow [7–9] and has served as the most reliable quantitative predictor of the success of resuscitation in experimental models and in human patients [10, 11]. Following the prolonged period of untreated cardiac arrest, the rationale of chest compression is to rapidly restore threshold levels of CPP and, therefore, myocardial blood flow. In the present study, all animals were submitted to identical qualities of external chest compressions and mechanical ventilations. The fact that CPP values during CPR were comparable between groups demonstrated that the qualities of chest compressions were well controlled for all animals. Our findings therefore suggested that the ease of defibrillation and resuscitability are not diminished by the preexisting chronic LV dysfunction but largely determined by the quality of CPR efforts.

Following ROSC, the primary goal of patient care is to ensure that the patient has adequate spontaneous circulation, such that the whole-body ischemia/reperfusion injury can be prevented, minimized, or reversed; subsequently, the ultimate postresuscitation survival with intact organ function might be improved. Reversible myocardial dysfunction has been observed after ROSC in experimental models [12] and in human patients [13, 14]. This dysfunction may result in acute hemodynamic compromise leading to profound hypoperfusion that adds additional ischemic injury to vital organs, and has been associated with early death after initial successful resuscitation [15]. In the present investigation, myocardial contractile dysfunction as assessed by decreased *dP*/*dt*_40_ and CI and diastolic dysfunction assessed by decreased −*dP*/*dt* were observed in all animals after resuscitation. Among these standard measurements, the index of resting CI represents global blood flow through the entire systemic circulation to vital organs. The positive correlation between CI and duration of postresuscitation survival has previously been demonstrated by us in a rodent model of VF/CPR [16, 17]. These observations indicated that inadequate systemic blood flow was associated with poor postresuscitation survival. In the present investigation, the fact that there was no difference in postresuscitation CI between groups suggested the comparable global organ blood flow after resuscitation. These may in part explain the insignificant difference in duration of postresuscitation survival between groups.

It is interesting to notice that no difference in CI following ROSC was observed between groups regardless of the significant difference before induction of VF. Previously the phenomenon of adaptive process of chronic hypoxia conferring myocardial tolerance to subsequent acute severe hypoxia/reoxygenation or ischemia/reperfusion injury has been observed in cardiac myocytes models [18, 19] and isolated perfused heart models [20]. Such observations have been supported by clinical investigation of coronary artery bypass surgery [21], in which investigators found that similar severity of ischemia/reperfusion induced moderate overall ultrastructural changes in normally contracting myocardium whereas only minor overall ultrastructural changes in postreperfusion hibernating myocardium. Similar to these observations, our work suggested that chronic heart failure might increase myocardial ischemic tolerance against impending insult of VF/CPR, which was manifested as the insignificant difference in postresuscitation CI, and subsequently the insignificant difference in postresuscitation survival between groups.

There are limitations that need to be acknowledged and addressed regarding the present study. Clinically, most episodes of VF are caused by ischemic heart disease rather than electric shock. We recognized that despite the minimal level of current flow, the potential electrical injury to the myocardium would likely compromise the clinical relevance of this rat model of VF/CPR. Regardless of this potential shortcoming, this, however, did not alter our conclusion since all the animals receive the same procedure. In this preliminary study, CPP is adopted as a reflection of myocardial blood flow. We admitted that ideally myocardial blood flow should be measured by real-time techniques during CPR and following resuscitation. However, aiming to observe the effect of chronic ischemic LV dysfunction on the duration of postresuscitation survival and the technical difficulties of directly measuring myocardial blood flow during chest compressions do not enable us to perform such measurement in real time. Nevertheless, based on the current data, we cautiously draw the conclusion that the extent of levels of CPP can, in part, reflect myocardial blood flow. In addition, we admit that our current studies lack direct evidence about the underlying mechanisms responsible for the phenomenon of “myocardial ischemic tolerance to insult of cardiac arrest and CPR.” This relatively preserved postresuscitation myocardial function in the chronic ischemic heart deserves further investigation. Finally, we admit that LVEF values before induction of VF are greater than those we usually observed in patients with chronic heart failure. Nevertheless, we do observe that the outcomes of CPR were not compromised by preexisting chronic ischemic LV dysfunction. The appropriate animal model and optimal experimental design will be considered in our future investigations, so that the effect of more depressed LVEF on the outcome of CPR can be further revealed.

Our findings may have potential clinical implications. First, our work indicates that efficacy of chest compressions during CPR overrides the detrimental effect of preexisting chronic heart failure in determining the likelihood of successful resuscitation. Second, since postresuscitation outcome was largely determined by the systemic blood flow, the postresuscitation patient care should be focused on maintaining and improving global blood flow following resuscitation, such that the ultimate postresuscitation survival with intact organ function might be improved.

## Figures and Tables

**Table 1:** Effects of intervention on ROSC, number of 72-hour survival, and number of defibrillations.

Group	ROSC	72-hour survival	Number of shocks
Chronic LV dysfunction	6/7	3/6	1.2 ± 0.4
Control	5/7	3/5	1.4 ± 0.9

Values are means ± SD. ROSC: return of spontaneous circulation.

## Abbreviations

CI: Cardiac index
CPP: Coronary perfusion pressure
CPR: Cardiopulmonary resuscitation
EF: Ejection fraction
LV: Left ventricular
LVDP: Left ventricular end-diastolic pressure
LVESV: Left ventricular end-systolic volume
LVEDV: Left ventricular end-diastolic volume
MAP: Mean aortic pressure
ROSC: Return of spontaneous circulation
VF: Ventricular fibrillation.

## References

[1] Shiga T, Hagiwara N, Ogawa H (2009). Sudden cardiac death and left ventricular ejection fraction during long-term follow-up after acute myocardial infarction in the primary percutaneous coronary intervention era: results from the HIJAMI-II registry. *Heart*.

[2] Bigger JT, Fleiss JL, Kleiger R (1984). The relationships among ventricular arrhythmias, left ventricular dysfunction, and mortality in the 2 years after myocardial infarction. *Circulation*.

[3] Schoenenberger AW, Kobza R, Jamshidi P (2009). Sudden cardiac death in patients with silent myocardial ischemia after myocardial infarction (from the Swiss Interventional Study on Silent Ischemia Type II [SWISSI II]). *American Journal of Cardiology*.

[4] Fang X, Tang W, Sun S (2006). Cardiopulmonary resuscitation in a rat model of chronic myocardial ischemia. *Journal of Applied Physiology*.

[5] Cobb LA, Fahrenbruch CE, Walsh TR (1999). Influence of cardiopulmonary resuscitation prior to defibrillation in patients with out-of-hospital ventricular fibrillation. *Journal of the American Medical Association*.

[6] Wik L, Hansen TB, Fylling F (2003). Delaying defibrillation to give basic cardiopulmonary resuscitation to patients with out-of-hospital ventricular fibrillation: a randomized trial. *Journal of the American Medical Association*.

[7] Halperin HR, Paradis N, Ornato JP (2004). Cardiopulmonary resuscitation with a novel chest compression device in a porcine model of cardiac arrest: improved hemodynamics and mechanisms. *Journal of the American College of Cardiology*.

[8] Lindner KH, Prengel AW, Pfenninger EG (1995). Vasopressin improves vital organ blood flow during closed-chest cardiopulmonary resuscitation in pigs. *Circulation*.

[9] Luce JM, Rizk NA, Niskanen RA (1984). Regional blood flow during cardiopulmonary resuscitation in dogs. *Critical Care Medicine*.

[10] Paradis NA, Martin GB, Rivers EP (1990). Coronary perfusion pressure and the return of spontaneous circulation in human cardiopulmonary resuscitation. *Journal of the American Medical Association*.

[11] Kern KB, Ewy GA, Voorhees WD, Babbs CF, Tacker WA (1988). Myocardial perfusion pressure: a predictor of 24-hour survival during prolonged cardiac arrest in dogs. *Resuscitation*.

[12] Kern KB, Hilwig RW, Rhee KH, Berg RA (1996). Myocardial dysfunction after resuscitation from cardiac arrest: an example of global myocardial stunning. *Journal of the American College of Cardiology*.

[13] Laurent I, Monchi M, Chiche J-D (2002). Reversible myocardial dysfunction in survivors of out-of-hospital cardiac arrest. *Journal of the American College of Cardiology*.

[14] Ruiz-Bailén M, Aguayo De Hoyos E, Ruiz-Navarro S (2005). Reversible myocardial dysfunction after cardiopulmonary resuscitation. *Resuscitation*.

[15] Brown CG, Martin DR, Pepe PE (1992). A comparison of standard-dose and high-dose epinephrine in cardiac arrest outside the hospital. *New England Journal of Medicine*.

[16] Sun S, Weil MH, Tang W, Kamohara T, Klouche K (2004). *δ*-opioid receptor agonist reduces severity of postresuscitation myocardial dysfunction. *American Journal of Physiology—Heart and Circulatory Physiology*.

[17] Tang W, Weil MH, Sun S, Pernat A, Mason E (2000). K(ATP) channel activation reduces the severity of postresuscitation myocardial dysfunction. *American Journal of Physiology—Heart and Circulatory Physiology*.

[18] Silverman HS, Wei S-K, Haigney MCP, Ocampo CJ, Stern MD (1997). Myocyte adaptation to chronic hypoxia and development of tolerance to subsequent acute severe hypoxia. *Circulation Research*.

[19] Crawford RM, Jovanović S, Budas GR (2003). Chronic mild hypoxia protects heart-derived H9c2 cells against acute hypoxia/reoxygenation by regulating expression of the SUR2A subunit of the ATP-sensitive K+ channel. *Journal of Biological Chemistry*.

[20] Tajima M, Katayose D, Bessho M, Isoyama S (1994). Acute ischaemic preconditioning and chronic hypoxia independently increase myocardial tolerance to ischaemia. *Cardiovascular Research*.

[21] Milei J, Fraga CG, Grana DR, Ferreira R, Ambrosio G (2004). Ultrastructural evidence of increased tolerance of hibernating myocardium to cardioplegic ischemia-reperfusion injury. *Journal of the American College of Cardiology*.

